# Role of radiosynovectomy in the treatment of rheumatoid arthritis and hemophilic arthropathies

**DOI:** 10.2349/biij.3.4.e45

**Published:** 2007-10-01

**Authors:** BK Das

**Affiliations:** Department of Nuclear Medicine, Radiotherapy and Oncology, School of Medical Sciences, Universiti Sains Malaysia, Penang, Malaysia

**Keywords:** Radiosynovectomy, treatment of hemophilic arthropathies, radionuclides in rheumatoid arthritis

## Abstract

Radiosynovectomy is a novel method of treatment for several acute and chronic inflammatory joint disorders. A small amount of a beta-emitting radionuclide is injected into the affected joint delivering a radiation dose of 70 to 100 Gy to the synovia. The proliferative tissue is destroyed, secretion of fluid and accumulation of inflammation causing cellular compounds stops and the joint surfaces become fibrosed, providing long term symptom relief. The radionuclides are injected in colloidal form so that they remain in the synovium and are not transported by lymphatic vessels causing radiation exposure to other organs. Complete reduction of knee joint swelling has been seen in above 40% and pain relief in 88% of patients. Wrist, elbow, shoulder, ankle and hip joints showed significant improvement in 50-60% and restoration of normal function and long term pain relief has been achieved in about 70% of small finger joints. In hemophilic arthropathies complete cessation of bleeding in about 60% and improved mobility in 75% of patients has been reported.

## INTRODUCTION

Joint disorders are relatively common in any society. Among the various forms of arthritis such as rheumatoid arthritis, osteoarthritis, villonodular synovitis, hemophilic arthropathies, psoriatic arthritis, ankylosing spondylitis and gout, acute and chronic rheumatoid arthritis and hemophilic arthropathies are most common, causing long suffering from pain, deformities and disability. It has been estimated that nearly 30 million people in the South East Asian region suffer from rheumatoid arthritis alone [1]. Conventional long-term treatment with various combinations of drugs can bring relief in many cases. However, some joints need additional local therapy. Mostly corticosteroids are injected into the joints to suppress the inflammatory process localised in the synovium.

In some cases, surgery is also performed. Radiosynovectomy (also known as radiosynoviorthesis) is a novel method of treatment for such joints. The concept of radiosynovectomy was reported earlier, but Delbarre *et al*. in the year 1968 [2] introduced the term ‘radio-synoviorthesis’ for the first time and also reported their clinical experience. Radisynovectomy or radiosynoviorthesis is defined as the restoration of inflamed and damaged synovial membrane of the joints after application of radionuclides (radioisotopes). The beta rays emitted by the radionuclides are used to effectively control the inflammatory process of the synovial membrane and the technique is indicated as an alternative therapy to early surgical synovectomy.

The purpose of this article is to describe the method and assess the impact of this novel technique in the overall management of rheumatoid arthritis and hemophilic arthropathies.

## MAGNITUDE OF THE PROBLEM

Rheumatoid arthritis (RA) is the most common chronic inflammatory disease of joints. It affect more than 1% of the population. This disease is more prevalent in women with a preponderance of 3:1 over men. It has been estimated that in Malaysia 0.22 million patients are suffering from this disease. It usually starts in fourth and fifth decade of life. However no age group in spared from this crippling disease. 80% of patients developing the disease are found to be between 35 to 50 years of age.

The causes of RA are not known, but there is strong evidence of involvement of cellular and humoral immune systems. This may be genetically predetermined as the incidence of sero-positive RA is more common in the population with HLA-DR4 (60%) than in normal controls (15%). It has also been suggested that RA manifests in response to certain environmental factors, and hormonal disturbances may play a role but the actual mechanism is not known. In many cases RA shows a “tumour like proliferation” with rapidly growing synovial membrane and pannus formation which behaves similar to a locally invasive tumour.

Clinically rheumatoid arthritis presents like a chronic multi-system disease with a variety of systemic manifestations but a characteristic feature is the persistent inflammatory synovitis. Most commonly involved joints are proximal inter-phalangeal joints, metacarpophalangeal joints, wrist and knee joints. In the long run synovial inflammation causes cartilage destruction, bone erosion and joint deformity with marked functional impairment.

Hemophilia is a common bleeding disorder in South East Asian countries. The incidence is more than 0.01% . More than half of the patients with this disorder suffer from arthropathy which cripples life at an early age.

The cause of hemophilia is a deficiency of clotting factors VIII (Hemophilia A) and IX (Hemophilia B). Inadequate replacement of factor VIII and IX, lack of patient education, lack of physician education regarding simple techniques (application of ice or ice packs, immobilisation of affected joints, use of slings), lack of physiotherapy and lack of new therapy methods like radiation synovectomy have contributed to the fact that more than 50% of these patients suffer from physical disability and crippling arthropathy.

## PRINCIPLES OF THE THERAPY

Radioactive isotopes which emit beta rays are used for Radiosynovectomy. The radionuclides in the form of colloids, on reaching the joint cavity are recognised as foreign bodies by the outermost cellular layer of the synovial membrane and are phagocytosed by these cells. Autoradiographic investigations show that Yttrium-colloids quickly enter the superficial layers and also to some extent the deeper layers of the synovial membrane but very little reaches the bones [3, 4]. Due to the selective radiation of the synovial membrane, there is necrosis of the cells and reduction in the inflammatory cellular proliferation. Arthroscopic examination shows a reduction in the number and size of the synovial villi and reduction in the hyperemic reaction [5, 6]. Later there is progressive fibrosis of the synovial stroma, the vessels and rarely, mild diffuse damage to the bones of the joint [7]. There is also prevention of the filtration and reabsorption of the synovial fluid.

There is complete disappearance of the mononuclear infiltration in the synovial membrane after a few months and the synovial membrane is fibrosed [8]. Further destruction of the joint cavity otherwise caused by continuous immunological reactions is prevented. Since the fibrotic tissue replacing the synovial membrane cannot react to the immunological stimulation, there is no recurrence of the inflammatory process and a long term remission is achieved.

## INDICATIONS

The basic understanding of treatment of rheumatoid arthritis is that it is a systemic disease and therefore, should be treated systemically. Radiosynovectomy is indicated when disease modifying therapy has been given for 6 months and in spite of that some joints are still affected and any increase of drug dosage may have serious side effects on the patient. Any exception from these basic principles of treatment can be made only by an expert in the field and in consultation with rheumatologist, orthopedician or the surgical rheumatologist. However, if radiosynovectomy is performed early in course of the disease, the overall result and prognosis of the treated joint appears to be better. Although best results are obtained in Steinbrocker stage I and II of the polyarthritis [9], radiosynovectomy is effective even in the later stages of the disease. The main indications for radiosynovectomy are: acute rheumatoid arthritis, chronic polyarthritis, psoriatic arthritis, anklylosing spondylitis with peripheral joint involvement, pigmented villonodular synovitis (6 weeks after surgery) haemarthrosis in hemophilia (not during active bleeding phase) and activated arthrosis or osteoarthritis.

## RADIONUCLIDES USED IN RADIOSYNOVECTOMY

In early studies radionuclide gold-198 in colloidal form was used. However, due to its gamma component leading to unwanted whole body radiation exposure combined with spread to lymph nodes and liver, it has been almost abandoned. There are a variety of radionuclides available now which are suitable for radiosynovectomy. The type of radionuclide to be used is determined by the size of the joint to be treated. The lesser range (weaker) beta rays are used in smaller joints like Erbium-169 for finger joints. Similarly the medium range beta rays of Rhenium-186, Phosphorus-32, etc. are used for larger joints (wrist, elbow, shoulder, ankle and hip joints) while the high energy beta rays of Yttrium-90 with tissue penetration of 3 to 11 mm are used for knee joints. In recent years several other radionuclides like Holmium-166, Rhenium-188, Samarium-153, etc. have successfully been introduced [10-18].

## DOSE CONSIDERATIONS

It is difficult to exactly determine the required dosage. The absorbed dose is not only dependent on the type of the radionuclide and the amount of activity (MBq / mCi) used but also on various other factors like the size of joint cavity, synovial thickness, distribution of the colloids in the joint fluid (water, gelatinous or hemorrhagic) and the inflammatory activity of the joints. A typical dose of 185 MBq (5mCi) of Yttrium (Yt-90) is used for knee joints. Approximately 100 Gy per 100 gm synovial tissue should be absorbed, to have optimal effect.

## SIDE EFFECTS

If done properly, no side effects have been observed. Infection of the joint is very rare (one in 35,000) in comparison to intra-articular corticosteroid injections. This is because of the intense beta radiation emitted by the high concentration of radioactive material in the joint killing all the bacteria. Temporary radiation or crystal synovitis, thrombosis due to immobilisation and lymphoedema may occur.

## PRACTICAL GUIDELINES

Ideally there should be close cooperation with the nuclear medicine physician, rheumatologist and orthopedician who would consider the indication and refer the patients for the procedure. It should be ensured that the basic therapy for rheumatic disease has been given for 6 months and surgical options for early or late synovectomy, tendon reconstruction (in case of tendon rupture), nerve decompression, etc. have been taken into consideration.

After taking the history and careful clinical examination of the patient, the risks, side effects and possible complications of the procedure have to be explained to the patient and a written consent has to be obtained.

In addition to the X-ray films of the affected joints, two more investigations, namely arthrosonography and scintigraphy, may also be considered to obtain optimal results.

## ARTHROSONOGRAPHY

It is sometimes possible to miss the presence of problematic Baker’s cyst of the knee joint during the clinical examination. If radiosynovectomy of the knee joint with Baker’s cyst is performed, it may lead to rupture of the cyst as a consequence of inflammatory reaction [19]. This is a fatal complication and must be avoided. As such Baker’s cyst of the knee joint with high risk of rupture is taken as a contraindication for radiosynovectomy. Fortunately Baker’s cyst can be definitely diagnosed by arthrosonography. In a study of 980 cases of knee joints treated by radiosynovectomy, Baker’s cyst was detected in 25% of the knee joints [20]. In the presence of a firm painful Baker’s cyst it is advisable to aspirate the cyst under ultrasound guidance and instill cortisone at least three days prior to radiosynovectomy. So arthrosonography is absolutely necessary prior to radiosynovectomy of the knee joints. But it may also be required in case of shoulder joint because it provides information of the condition inside the joint (effusion, rotator cuff rupture of the shoulder, sub deltoid bursitis etc.) and also about the peripheral structures (tenosynovitis, enthesitis etc.). These details and use of an intensifier help to increase the accuracy of intra articular administration of the radiopharmaceutical.

## JOINT SCINTIGRAPHY

The usual skeletal scintigraphy is performed 3 hours after injection of the radiopharmaceutical (usually 99m-Tc MDP / HDP). In soft tissue scintigraphy, images have to be taken 5 minutes post injection at a time when most of the radiopharmaceutical is still in the blood stream and in soft tissues. The hyperemic areas in synovitis seen as increased concentration of the radiopharmaceutical are highly suggestive of inflammatory activity at the joint. This procedure gives fairly accurate information about the polyarticular involvement and the intensity of synovitis which correlate well with the degree and the intensity of pain, even months before any radiological changes are seen in the joint. For example, it is often possible to identify the particular tendons or joints of the middle foot causing the actual pain to the patient who sometimes may not be able to pinpoint exactly the site of involvement. The soft tissue scintigraphic images also help to identify the suitable joints, which will respond to radiosynovectomy [21].

## DOSE INJECTION

Since even a slight extra- articular extravasation of the radioactive material can lead to tissue necrosis, it is very important to be perfect in the injection technique. All joints with the exception of knee joints must be injected only under radiological guidance. A x-ray machine called C-arm which is otherwise used for various radiological procedures can be used for the purpose.

In all joints (specially the small finger joints) arthrographic orientation of the joint space and the ideal position for the needle placement should be done before starting the procedure.

After the procedure a distribution scintigram of the joint wherever possible may be performed to document the distribution of the radionuclide in the joint.

It is advisable to inject a corticosteroid preparation along with the radio nuclide for following benefits:

- Radiation synovitis with effusion (knee joint) can be avoided.
- The inflammatory component will subside effectively through the corticosteroid so that the radionuclide therapy can be more effective.
- In the wrist joint, it helps Rhenium (Rh-186) to spread well in the distally located inter carpal compartments.
- It helps to relieve the patients of the symptoms immediately and thereby bridge the time till effects of intense radiation from the radionuclide sets in.

After the radiosynovectomy, the joint should be immobilised for 48 hours with help of a splint. It will help to prevent the lymphatic spread and reduce leakage rate if the joint is given rest for about a week [22].

It takes up to 3 months to get the full effects of radiosynovectomy. Symptoms may persist during this period and sometimes there may also be an effusion which needs to be drained during the follow up after 3 or 4 months. If corticosteroid has been injected simultaneously, patient may remain free of complaints even during the initial period till the radiosynovectomy becomes effective.

## RESULT AND DISCUSSION

A large number of procedures using Yittrium-90 for knee joints have been performed worldwide. Improvement rates ranging from 40 to 100% have been reported [23-27]. In two years follow up of patients, knee joint swellings were reduced almost completely in 38%, pain relief was achieved in 88% and stretching deficiency corrected in 71% of the cases [22]. Some reports quote an improvement rate of 85% after 3-4 years [25]. Rhenium (Re-186) is used for middle sized joints. Good to excellent results are seen in 60-80% of the cases in hand, elbow, shoulder, ankle and hip joints [28]. Good to very good results have been reported in 83% of the elbow joints [29, 30].

Erbium (Er-169) is used in small joints of the fingers. Good to very good results leading to restoration of normal function has been reported in 54.6% in a study consisting 1261 finger joints [31, 32].

Similar results were reported by Boussiana *et al.* in a double blind study conducted involving PIP joints in 35 patients suffering from chronic polyarthritis who did not respond to intra articular corticosteroid injections [32]. Good to excellent results were seen in 71.5% of cases in relation to pain relief and joint mobility 6 months after radiosynovectomy. After one year good to excellent results were seen in 79.4%, with no improvement in 20.6% of cases. The X-ray findings of the joints treated by radiosynovectomy showed no changes after 1 year as against those in placebo group (physiological NaCl) which showed reduction in the joint space. In addition, there was fibrosis and absence of inflammatory changes in those joints treated with Er-169 whereas persistence of all histological changes of rheumatoid arthritis was seen in the placebo group [33].

Since the radionuclides are applied in form of colloids of appropriate size, they remain mostly within the joints. There is no significant radiation exposure to other organ or parts of the body. Being beta emitters, they also don’t pose any radiation to the environment, so the procedure can be performed on ambulatory patients.

If adequate response has not been achieved or recurrence of the disease occurs it may be advisable to repeat the procedure, preferably 6 months after the first treatment. Favourable results have been reported after re-radiosynovectomy of the knee joints in cases of rheumatoid arthritis.

## CONCLUSION

Radiosynovectomy is an effective alternative therapeutic approach in many conditions needing additional treatment of individual joints. It has been found to be cost effective in providing long term relief of pain and deformity.

The number of centres performing radiosynovectomy is increasing all over the world and reports of new indications like application in complication of total knee joint prosthesis leading to effusion is increasingly seen in literature [34]. There is no radiation risk and the procedure can be performed on an outpatient basis.
